# A Case of an Interventricular Septum Pseudoaneurysm With Perforation Mimicking a Ventricular Septal Defect

**DOI:** 10.7759/cureus.73080

**Published:** 2024-11-05

**Authors:** Dawood Jamil, Raef Fadel, Patrick Kollman, Benjamin Swanson

**Affiliations:** 1 Internal Medicine, Henry Ford Health System, Detroit, USA; 2 Cardiology, Henry Ford Health System, Detroit, USA; 3 Internal Medicine, Wayne State University School of Medicine, Detroit, USA

**Keywords:** cardiothoracic and vascular surgery, critical care cardiology, intervention cardiology, ventricular pseudoaneurysm, ventricular septal defect (vsd)

## Abstract

Ventricular pseudoaneurysm (PSA) is a ventricular outpouching contained by adherent pericardium or myocardial scar tissue and represents a rare but potentially fatal complication of acute myocardial infarction (AMI). The vast majority of cases involve the left ventricular apex, in the area of infarct. It is extremely rare to see PSA formation within the interventricular septum (IVS). We present a case of ventricular PSA of the IVS, with contained perforation into the right ventricle, mimicking a ventricular septal defect (VSD) in a patient presenting with ST-elevation myocardial infarction (STEMI). This case underscores the importance of maintaining a high index of clinical suspicion and reviews the pathophysiological mechanisms and treatment options for these life-threatening mechanical complications.

## Introduction

With the advent of coronary reperfusion therapies, the incidence of structural pathology post-myocardial infarction (MI) has decreased significantly. However, when these complications do occur, the associated mortality remains high [1]. Among these complications, ventricular septal defect (VSD) formation remains the most common and can occur in roughly 0.21% of patients with ST-elevation myocardial infarction (STEMI) [1]. Unlike VSDs, ventricular pseudoaneurysms (PSAs) are contained by pericardium or scar tissue, thereby limiting the extravasation of blood outside the ventricle [2]. A study by Frances et al. demonstrated that 55% of ventricular PSAs were due to MI, of which 49% were inferior MI [3]. Additionally, a retrospective analysis, done between 1980 and 1996 at the Mayo Clinic, evaluated 52 patients with cardiac PSA and determined that the majority (82%) who presented with MI had PSA in the inferior or posterolateral walls [4].

Interventricular septum (IVS) PSA is a rare entity, with few cases reported in the literature to date. In this case, the PSA was discovered on transesophageal echocardiogram (TEE) and confirmed during open heart surgery for both the repair of the perforation and coronary artery bypass grafting (CABG). Three similar cases have been reported in the literature involving PSA of the IVS, two of which were fatal.

## Case presentation

**Figure 1:**
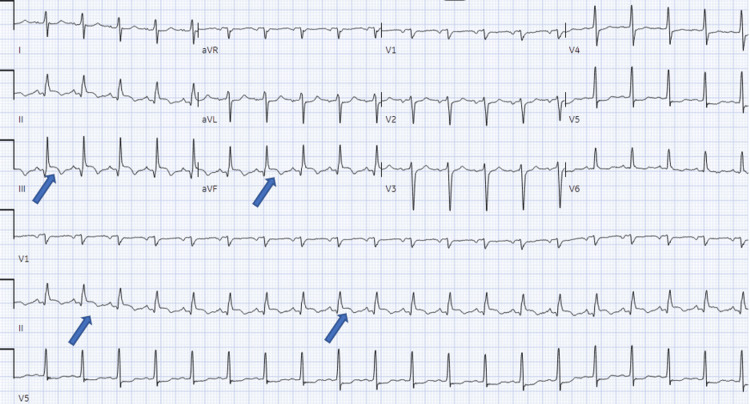
ECG on admission, demonstrating ST elevations in leads II, III, and aVF. ECG: Electrocardiogram; aVF: Augmented voltage foot; aVR: Augmented voltage right; aVL: Augmented voltage left

We present a case of a 62-year-old male with a past medical history significant for type 2 diabetes, hypertension, and hyperlipidemia, who presented to an outside hospital with a four-day history of exertional chest pain and shortness of breath. On admission, he was noted to have elevated troponin of 2307 ng/L (ref <4 ng/L), and his electrocardiogram (ECG) demonstrated ST elevations in the inferior leads (Figure 1).

**Figure 2:**
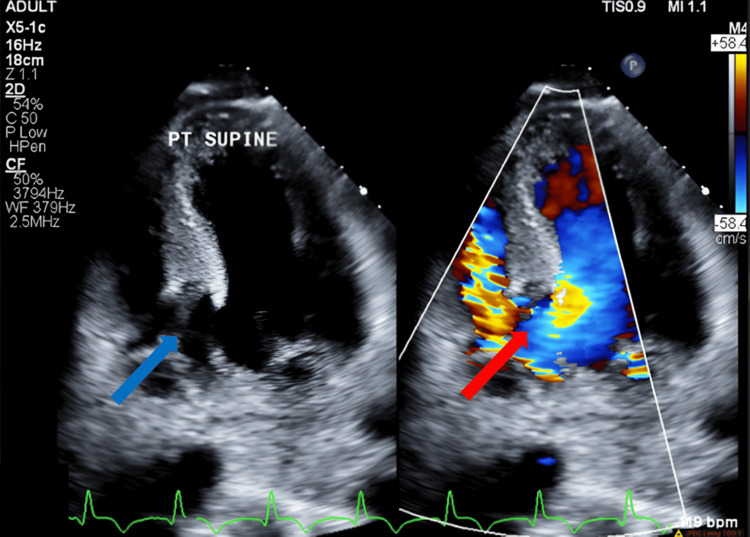
Apical four-chamber view, demonstrating the presence of muscular VSD (blue arrow) with Doppler demonstrating left-to-right shunting (red arrow). VSD: Ventricular septal defect

He was taken for an urgent left heart catheterization (LHC), which demonstrated multivessel coronary artery disease with no identifiable culprit lesion, which was not amenable to percutaneous coronary intervention. Post-cardiac catheterization, the patient was transferred to the intensive care unit, where he experienced worsening chest pain, oxygenation, and hemodynamics consistent with cardiogenic shock. A transthoracic echocardiogram (TTE) was performed, which demonstrated a large VSD with moderate left-to-right shunting (Figure 2) and showed a cardiac output of 10.98 L/min and an ejection fraction of 57%, prompting placement of an Impella with an initial setting of 3.8 L/min prior to transfer to our facility.

**Figure 3:**
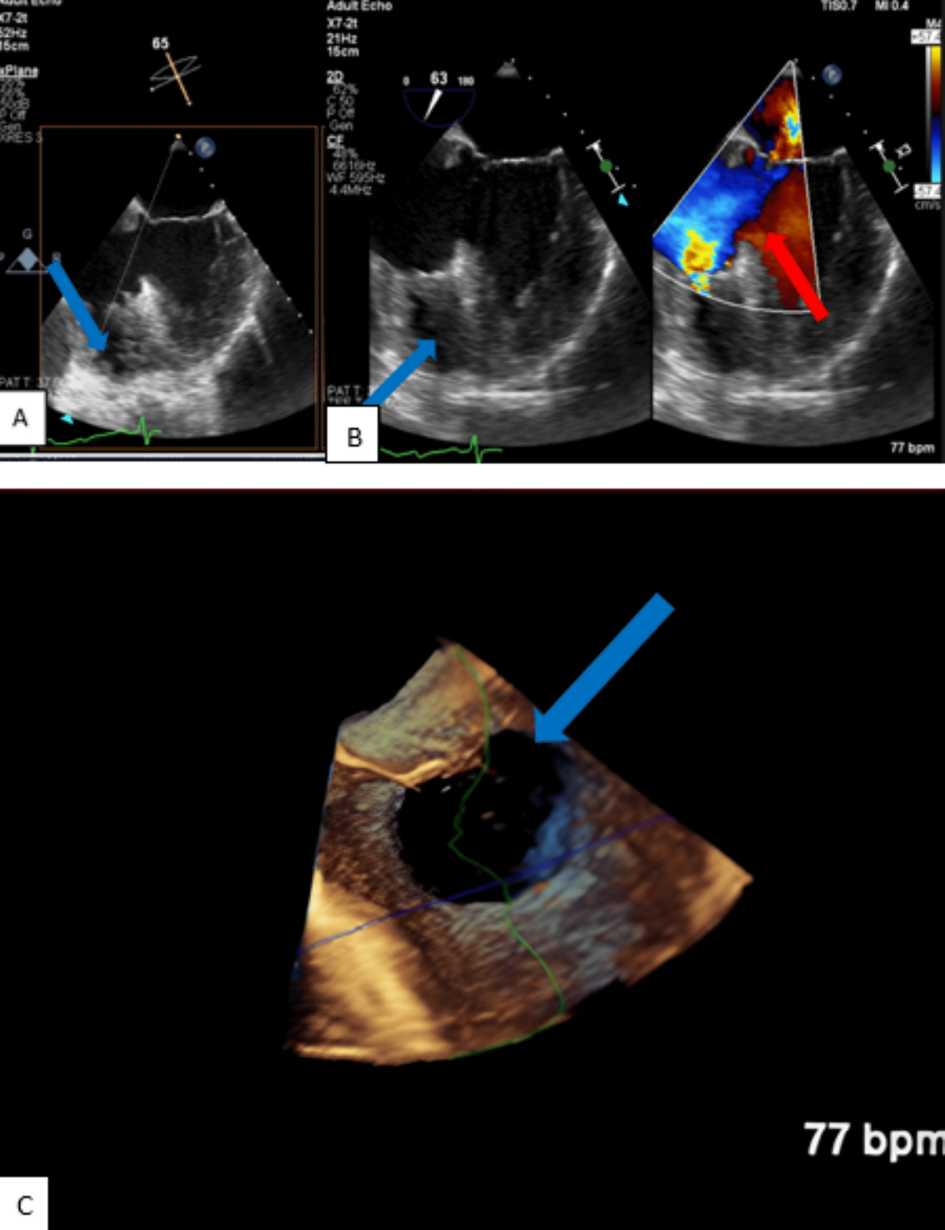
(A) TEE demonstrating an aneurysmal sac protruding into the right ventricle (blue arrow); (B) TEE demonstrating the presence of contained rupture into the right ventricle distal to the tricuspid annulus (blue arrow) with color doppler ultrasound revealing turbulent flow (red arrow); (C) Three-dimensional TEE revealing the 3.55 x 3.79 cm orifice (blue arrow). TEE: Transesophageal echocardiogram

On arrival, he was evaluated by cardiac surgery, who initially held off on acute surgical intervention, given the increased tissue fragility following an acute myocardial infarction (AMI). The patient’s Impella (Abiomed, Inc., Danvers, MA, USA) was removed, and he was upgraded to left atrial veno-arterial extracorporeal membrane oxygenation (LAVA-ECMO), peripherally placed via the right femoral artery, to facilitate myocardial tissue recovery. A bedside TEE demonstrated a large PSA with a 3.55 x 3.79 cm orifice within the IVS, with contained perforation into the right ventricle (Figure 3).

After 10 days on mechanical support, he was taken to the operating room (OR) and was noted to have a large posterior PSA with a 4 x 4 cm perforation into the right ventricle, correlating remarkably with measurements by TEE. He received two overlapping bovine pericardial patches and underwent a two-vessel CABG of the left main coronary artery. Post-operatively, he required an intra-aortic balloon pump to aid in weaning from cardiopulmonary bypass, along with inotropic and vasopressor support, which were gradually tapered off. The patient was ultimately discharged home with cardiac rehab and recently rejoined his competitive softball league.

## Discussion

Mechanical complications of AMI, such as VSDs, ventricular free wall rupture, papillary muscle rupture, and ventricular aneurysm/PSA formation, are rare but serious conditions that substantially increase in-hospital mortality following AMI [5]. The pathophysiology of these mechanical complications involves extended ischemia and necrosis of myocardial tissue, causing structural weakness or excessive scarring, resulting in rupture [6]. Early diagnosis and appropriate management of mechanical complications are essential to optimize the outcomes of patients post-AMI. The patient we present demonstrates a rare case of two concordant and interconnected mechanical complications: a ventricular PSA and a VSD.

VSDs are the most common mechanical complications, accounting for about 75% of these complications, according to a 2019 study by Elbadawi et al., who evaluated 13,767 patients with mechanical complications following AMI [7]. The magnitude of the left-to-right shunt created by the VSDs is an important predictor of mortality; specifically, the degree of the shunt influences the severity of subsequent cardiogenic shock and congestive heart failure [1]. Even in the absence of hemodynamic instability, a ventricular septal rupture should be suspected in the presence of a new systolic murmur at the left sternal border or by echocardiographic evidence of a left-to-right shunt and right ventricular volume overload [2]. Our patient experienced an acute inferior STEMI, leading to a posterior VSD with moderate left-to-right shunt seen on echocardiography, requiring surgery for definitive treatment. While an Impella device and then LAVA-ECMO were used for temporary circulatory support, surgical treatment was required for definitive treatment.

Ventricular PSAs/aneurysms, another mechanical complication of AMI, are outpouchings of the ventricular wall that either contain all layers of the myocardial wall (true aneurysm) or are ruptures of the wall contained by adherent pericardium (PSAs). PSAs often present subacutely, but true ventricular aneurysms typically present within two weeks of AMI [5]. Previously identified complications of ventricular aneurysms include thrombus formation, angina, and heart failure [5].

Various treatment options are employed for mechanical complications of AMI, with different interventions indicated for varying conditions and severities. While medical therapy and mechanical cardiopulmonary support are often critical for the stabilization of patients with these complications, surgery is usually preferred or required for long-term treatment [6]. Surgical closure is the typical standard treatment for VSDs after AMI [8]. The surgical technique typically involves suturing a pericardial or prosthetic patch over the VSDs, anchored to adjacent healthy myocardium [6]. However, some studies have shown that small-to-medium-sized VSDs can be amenable to percutaneous intervention with similar outcomes, while larger VSDs require surgical intervention [8]. True aneurysms and PSAs often require surgery for treatment, with limited percutaneous treatment options [8]. Surgical indications for true aneurysms are typically based on the severity of symptoms and the potential for complications, such as rupture, while PSAs have a higher tendency for growth or rupture and usually require intervention [6].

## Conclusions

The case highlights a rare complication of AMI with a unique presentation and diagnostic dilemma. While a VSD was detected on TTE, findings on TEE and in the OR confirmed a ruptured interventricular PSA. This rare complication highlights the challenge of precisely diagnosing these conditions on both TTE and TEE. Such cases present unique challenges in the diagnosis and treatment of this dangerous complication of AMI, given the requirement for multi-modal imaging and, ultimately, cardiac surgery for definitive diagnosis and management. Ultimately, with strategically delayed surgical intervention, our patient underwent successful definitive management in the OR, resulting in an excellent outcome.
